# The strength of the HIV-1 3' splice sites affects Rev function

**DOI:** 10.1186/1742-4690-3-89

**Published:** 2006-12-04

**Authors:** Susanne Kammler, Marianne Otte, Ilona Hauber, Jørgen Kjems, Joachim Hauber, Heiner Schaal

**Affiliations:** 1 Institut für Virologie, Heinrich-Heine-Universität Düsseldorf, Universitätsstr. 1, Geb. 22.21, D-40225 Düsseldorf, Germany; 2 Heinrich-Pette-Institute for Experimental Virology and Immunology, Martinistrasse 52, D-20251 Hamburg, Germany; 3 Department of Molecular Biology, University of Aarhus, C.F. Møllers Allé, Bldg. 1130, DK-8000 Aarhus C, Denmark; 4 Institut für Genetik, Heinrich-Heine-Universität Düsseldorf, Universitätsstr. 1, Geb. 26.03, D-40225 Düsseldorf, Germany

## Abstract

**Background:**

The HIV-1 Rev protein is a key component in the early to late switch in HIV-1 splicing from early intronless (e.g. *tat*, *rev*) to late intron-containing Rev-dependent (e.g. *gag*, *vif*, *env*) transcripts. Previous results suggested that *cis*-acting sequences and inefficient 5' and 3' splice sites are a prerequisite for Rev function. However, we and other groups have shown that two of the HIV-1 5' splice sites, D1 and D4, are efficiently used *in vitro *and *in vivo*. Here, we focus on the efficiency of the HIV-1 3' splice sites taking into consideration to what extent their intrinsic efficiencies are modulated by their downstream *cis*-acting exonic sequences. Furthermore, we delineate their role in RNA stabilization and Rev function.

**Results:**

In the presence of an efficient upstream 5' splice site the integrity of the 3' splice site is not essential for Rev function whereas an efficient 3' splice site impairs Rev function. The detrimental effect of a strong 3' splice site on the amount of Rev-dependent intron-containing HIV-1 glycoprotein coding (*env*) mRNA is not compensatable by weakening the strength of the upstream 5' splice site. Swapping the HIV-1 3' splice sites in an RRE-containing minigene, we found a 3' splice site usage which was variably dependent on the presence of the usual downstream exonic sequence. The most evident activation of 3' splice site usage by its usual downstream exonic sequence was observed for 3' splice site A1 which was turned from an intrinsic very weak 3' splice site into the most active 3' splice site, even abolishing Rev activity. Performing pull-down experiments with nuclear extracts of HeLa cells we identified a novel ASF/SF2-dependent exonic splicing enhancer (ESE) within HIV-1 exon 2 consisting of a heptameric sequence motif occurring twice (M1 and M2) within this short non-coding leader exon. Single point mutation of M1 within an infectious molecular clone is detrimental for HIV-1 exon 2 recognition without affecting Rev-dependent *vif *expression.

**Conclusion:**

Under the conditions of our assay, the rate limiting step of retroviral splicing, competing with Rev function, seems to be exclusively determined by the functional strength of the 3' splice site. The bipartite ASF/SF2-dependent ESE within HIV-1 exon 2 supports cross-talk between splice site pairs across exon 2 (exon definition) which is incompatible with processing of the intron-containing *vif *mRNA. We propose that Rev mediates a switch from exon to intron definition necessary for the expression of all intron-containing mRNAs.

## Background

**Figure 1:**
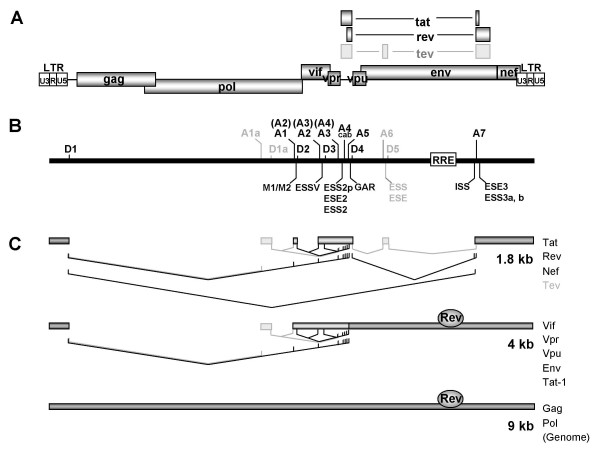
**Alternative splicing of HIV-1**. **(A) **Organization of the HIV-1 genome. Filled boxes indicate open reading frames present in all isolates, light grey boxes indicate the Tev orf which is isolate specific. The long terminal repeats (LTR) are present at both ends of the proviral DNA. **(B) **Localization of splice sites, splicing regulatory elements and the Rev responsive element (RRE). 5' splice sites: D1a-5; 3' splice sites: A1-7. Splice sites A6/D5 are isolate specific and not functional in the isolate NL4/3 used in this study. Splice sites A1a/D1a defining exon 1a have been recently described [6]. The nomenclature of the 3'ss is according to Stoltzfus [17,18] and Purcell and Martin [2] (in brackets). Splicing regulatory elements: M1, M2 (this report); ESSV [16,64]; ESS2p [18]; ESE2/ESS2 [17,32,43,44,49]; GAR [23,28]; ESS/ESE [19,20]; ISS [22]; ESE3 [17,21,24,25,33,65]; ESS3a, b [17,21,24,33,66]. **(C) **Splicing pattern and proteins encoded by the different mRNA classes. The 1.8 and 4 kb mRNAs contain obligatory sequences (dark grey) as well as alternative sequences (light grey) due to alternative usage of the splice sites. The nuclear export of the 4 kb mRNAs and the genomic full-length 9 kb mRNA is dependent on Rev binding.

During replication of the human immunodeficiency virus type 1 (HIV-1) the viral (+)RNA genome is reverse transcribed and integrated into the host cell genome. Transcription of this provirus by the cellular RNA polymerase II generates a polycistronic pre-mRNA that contains at least four 5' splice sites (5'ss) D1-4 and eight 3' splice sites (3'ss) A1, 2, 3, 4c, 4a, 4b, 5 and 7 that enable alternative splicing of more than 40 different mRNAs. Additionally, isolate specific (D5 and A6) and subgenomic construct-specific usage of cryptic splice sites has also been reported [1-4] (for a recent review see [5] and Fig. 1). Beside these well-known 5'ss, additional sites might be present preferentially serving as U1 snRNA binding sites to stabilize the viral RNA rather than serving for transcript diversity (e.g., 5'ss of exon 1a, [6]).

Replication of HIV-1 requires an early to late switch in splicing from early intronless to late intron-containing Rev-dependent mRNAs. The intronless transcripts of the 1.8-kb or "multiply spliced" class code for the regulatory and accessory proteins Tat, Rev and Nef. Processing of these transcripts is fully compatible with the model of exon recognition. In the late phase, all transcripts of the 4.0-kb class coding for the Env, Vpu, Tat, Vpr and Vif proteins contain at least one intronic sequence. However, due to the variable inclusion of the small non-coding leader exons 2 and/or 3 in some cases these so-called "partially or incompletely spliced" mRNAs are even more often spliced than the early "multiply spliced" Rev-independent mRNAs (cf., the "partially or incompletely spliced" 1.2.3.5I *env *RNA vs. the "multiply spliced" Rev-independent 1.7 *nef *mRNA). Thus, the number of intron removals is not decisive for Rev-dependence but rather the implementation of intron definition.

Whereas the early 1.8-kb mRNA species readily exit the nucleus and undergo translation, the 4.0-kb and non-spliced 9.0-kb mRNAs require Rev which overcomes the restriction of nuclear export of intron-containing transcripts by accessing the CRM1 nuclear export pathway [7,8]. In particular, the viral transcripts encoding the Env, Vpu, Vpr, Vif and structural viral Gag, Gag-Pro, and Gag-Pro-Pol proteins include the *tat/rev *intron flanked by D4 and A7, which contains a complex secondary structure, i.e., the Rev response element (RRE) which functions as high-affinity binding site for Rev.

Even though the interactions between splicing and Rev-dependent mRNA export are still not totally understood it is commonly accepted that *cis*-acting sequences in *gag*/*pol *and *env *[9-11], as well as inefficient splice sites [12,13], are prerequisites for the Rev-regulated HIV-1 gene expression. In fact, based on their sequence-mediated intrinsic strength, the HIV-1 splice acceptors are predicted to be inefficient. They all contain suboptimal polypyrimidine tracts (PPTs) interrupted by purines and, in some cases, by other AG dinucleotides and branch point sequences (BPSs) with 1–4 mismatches to U2 snRNA. For A2, A4a, A5 and A7 even branching on uracil or guanine instead of the typically used adenine has been reported [14,15].

Determination of the strength of a splice site however, is exacerbated by the fact that its intrinsic strength can be greatly modified, both positively as well as negatively, by *cis*-acting splicing regulatory sequences called splicing enhancers and silencers. Several *cis*-acting elements i.e. splicing silencer elements, have been identified in the HIV-1 genome. These serve as protein binding sites for members of the heterogenous nuclear ribonucleoprotein (hnRNP) family by down regulating splicing at the 3' splice sites A2 [16], A3 [17,18], the HXB2-specific A6 [19,20] and A7 [17,21,22]. Interestingly, and also a priori unexpectedly for inefficient splice sites, previous studies have also mapped splicing enhancer sequences as binding sites for SR proteins in exon 5 [23], the HXB2 specific exon 6 [20] and downstream of A7 [17,21,22,24-26]. Binding of SR proteins downstream of a splice acceptor can increase the efficiency of U2AF binding to the polypyrimidine tract either by displacement of hnRNP A1 protein that blocks access of spliceosomal components to the 3'ss or by direct interaction between the RS domains of the SR protein and U2AF35.

Previous experimental studies examining the strength of HIV-1 3' splice sites support the predicted inefficiency of these sites but did not take into account the influence of all the *cis*-acting sequences which had not been identified at that time [27]. Therefore, we were interested in examining the impact of the intronic sequence versus the *cis*-acting, mostly exon-located, enhancer and silencer elements on the strength of the retroviral 3'ss.

In a splice site swapping strategy we compared the splicing efficiency of the HIV-1 3'ss A1, 2, 3, 4cab, 5 and 7 in the presence and absence of their natural downstream exonic sequences. Since HIV-1 exon 2 drastically increased usage of the intrinsic weak splice site A1, which is required for the *vif *mRNA, we characterized this newly identified bipartite ESE and show that inactivation of the heptameric sequence M1 within an infectious molecular clone specifically impedes exon definition.

## Results

### A functional 3' ss is not necessary for Rev function

**Figure 2:**
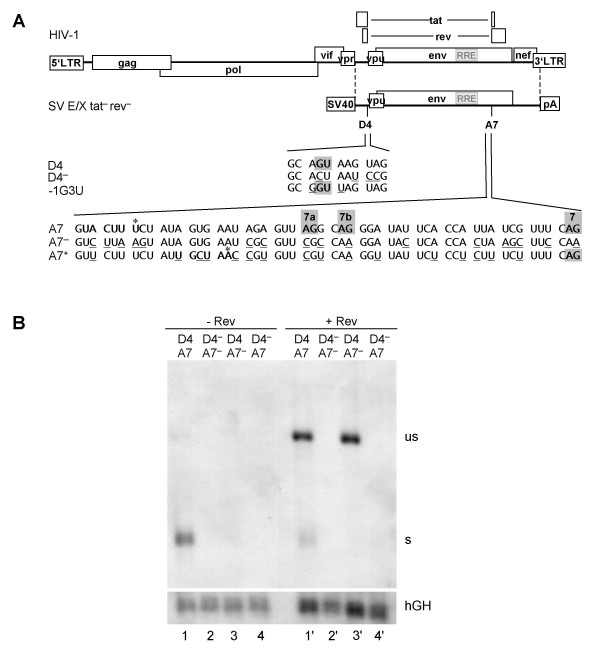
**3'ss A7 is nonessential for RNA stability and Rev responsiveness**. **(A) **Schematic drawing of the HIV-1 genome and of the subgenomic *env *expression plasmid SV E/X tat^- ^rev^-^. LTR: long terminal repeat, SV40: SV40early promoter, pA: SV40 polyadenylation sequence. Nucleotide sequences of the 5'ss D4 and its mutations D4^- ^and -1G3U as well as the 3'ss A7 and its mutations A7^- ^and A7^+ ^are shown beneath. The splice sites (grey squares, including the minor 3'ss 7a and 7b), the reported or supposed branch point sequence (bold, asterix indicates the branch point nucleotide) and the mutated nucleotides (underlined) are marked. In the 3'ss mutants the reading frame was kept unchanged except for position 703 (Val→Ala) in A7^+^. **(B) **HeLa-T4^+ ^cells were transiently transfected with the subgenomic *env *expression plasmids (SV E/X tat ^- ^rev ^-^) containing either the wild type 5'ss D4 or the non functional D4^- ^mutation combined with either the wild type 3'ss A7 or the A7^- ^mutation in presence or absence of a Rev expression plasmid (SVcrev) as indicated above the lanes. The poly(A)^+ ^RNA was analyzed by Northern blotting. s: spliced, us: unspliced transcript. Transfection efficiency was monitored by co-transfection of a human growth hormone (hGH) expressing plasmid (pXGH5).

Binding of U1 snRNA to 5'ss D4 within a subgenomic *env *mRNA has turned out to substantially increase the *env *mRNA steady-state level. Therefore, the presence of D4 not only has been a prerequisite for splicing but also for the nuclear export of unspliced RNA through the action of Rev. To analyze whether 3'ss A7 also contributes to the steady-state level of the glycoprotein mRNA we inactivated 3'ss A7 and the two upstream minor 3'ss, A7a and A7b [2-4], by silent point mutations (A7 ^-^, Fig. 2A) enabling analysis of these mutations in the glycoprotein-mediated syncytia assay.

To verify that the introduced mutations did not lead to activation of a cryptic 3'ss we additionally compared the subgenomic HIV-1 transcripts by Northern blot analysis of the respective poly(A)^+ ^RNA fractions following transient transfection of HeLa-T4^+ ^cells with either the subgenomic *env *expression vector SV E/X *tat*^- ^*rev*^- ^or SV E/X *tat*^- ^*rev*^- ^A7^- ^(Fig. 2B). Due to mutations of the *tat *and *rev *ATG translational initiation codons, these vectors express neither Tat nor Rev. Thus, in the absence of Rev transfection with SV E/X *tat*^- ^*rev*^- ^led almost exclusively to detection of spliced mRNA (Fig. 2B, lane 1). In contrast, after cotransfection with a Rev-expressing plasmid the majority of the detected mRNA was the unspliced poly(A)^+ ^glycoprotein mRNA (Fig. 2B, lane 1'). As expected, mutations of all three 3'ss, A7, A7a and A7b, led to complete loss of any detectable spliced transcript indicating that no cryptic splice acceptor was significantly activated (lane 3). However, in the presence of Rev the amount of unspliced poly (A)^+ ^*env *mRNA was unaffected by the presence or absence of a functional splice acceptor (cf. lane 1' with 3') demonstrating that the 3'ss mutations did not decrease the pool of unspliced poly (A)^+ ^transcripts. This contrasted the previously shown 5'ss dependency of spliced and unspliced transcripts [28] (cf. lane 1 with 4 and 1' with 4'), i.e. the lack of U1 snRNA-binding to the 5'ss leads to *env *RNA degradation (see hGH detectability in lanes 2, 4, 2', and 4'). The results of the Northern analysis were confirmed by glycoprotein expression analyzed by Western blot and syncytium formation (data not shown). Together these results demonstrate that a 3'ss is dispensable for Rev-mediated *env *expression. Moreover, these results show that the protective function of U1 snRNA binding is independent of the recognition of the 3'ss during progression of spliceosome formation.

**Figure 3:**
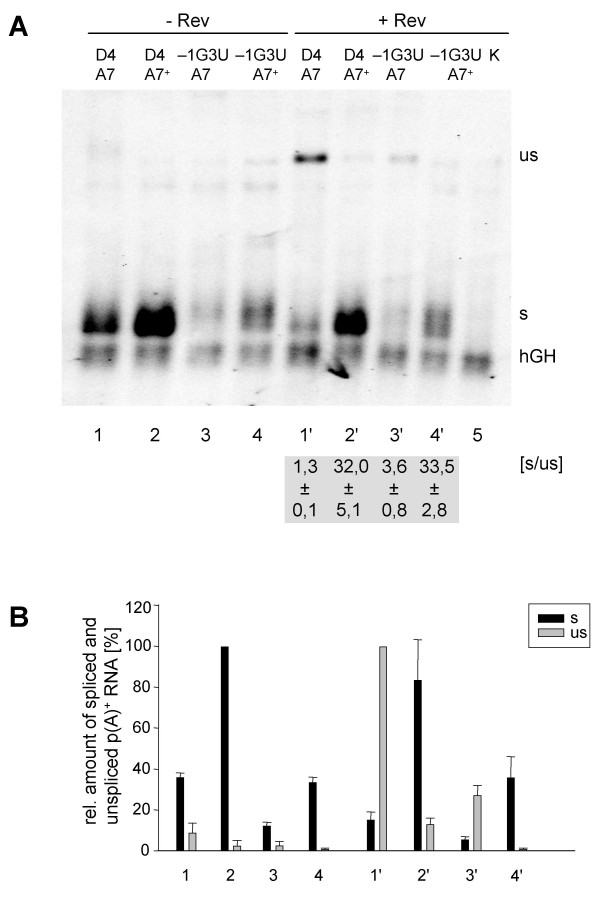
**Weakening of the 5'ss D4 does not compensate for the strength of 3'ss A7**. HeLa-T4^+ ^cells were transiently transfected with the subgenomic HIV-1 constructs (SV E/X tat ^- ^rev ^-^) combining an efficient (A7^+^) or inefficient (A7) 3'ss with a 5'ss with high (D4) or lower (-1G3U, cf. Fig. 2A) complementarity to U1 snRNA. The p(A)^+ ^RNA was analyzed by Northern blotting (cf. Fig. 2). **(A) **Northern blot with indication of the ratio of spliced (s) and unspliced (us) RNA in presence of Rev ([s/us], mean ± standard error) from three independent experiments. **(B) **Mean values of the relative amounts of spliced (s, black) and unspliced (us, grey) transcripts from three independent experiments, normalized to transcription efficiency (hGH). The spliced (s) and unspliceds (us) RNA populations were quantified from different exposure times of the blots to adjust for the different levels of signal intensities. The maximum values of both RNA populations were defined as 100%.

To exclude the possibility that the requirement for U1 snRNA complementarity for protection of the transcript was caused by an RNA surveillance mechanism detecting a functional 3'ss in the absence of a 5'ss we mutated both the 5'ss (D4^-^, Fig. 2A) and 3'ss (A7^-^) and analyzed the steady-state levels of total poly(A)^+ ^RNA. In the absence of both the 5' and 3'ss RNA could still not be detected irrespective of the presence or absence of Rev (Fig. 2B, lanes 2 and 2'). This indicates that the U1 snRNA dependency for the expression of this subgenomic *env *mRNA was not due to an unpaired/cryptic splice site but was intrinsic to the transcript sequence.

### 3'ss efficiency competes with Rev function

Increasing the complementarity between the 5'ss D4 and U1 snRNA did not lead to a decrease in *env *expression, indicating that even in the presence of a strong 5'ss Rev-regulated *env *mRNA transport was not impaired [23,29]. To specifically investigate the influence of the strength of the 3'ss on Rev-mediated glycoprotein expression we improved the strength of 3'ss A7 in its context of a subgenomic glycoprotein expression vector. To achieve this, the suboptimal BPS was attenuated and a new BPS with higher complementarity to U2 snRNA was created further downstream. Additionally, the canonical AG dinucleotides of the cryptic sites A7a and A7b were mutated to prevent an interference with potentially binding splicing factors and the pyrimidine content of the PPT (in the region between the new BPS and the intron/exon border) was increased from 48 % to 77 %. All these nucleotide changes were introduced as silent mutations except for one (Val to Ala at position 703), which was not expected to influence the fusogenic activity of the glycoprotein (Fig. 2A, A7^+^).

As expected, analysis of HeLa-T4^+ ^cells transfected with this vector revealed that the introduced mutations improved the efficiency of A7 as evident by a dramatic increase in the amount of spliced transcript (Fig. 3A and 3B, cf. lane 1 with 2). This was also confirmed by *in vitro *splicing experiments with the respective splicing constructs (data not shown). In the presence of Rev however, almost no unspliced poly(A)^+ ^message was observed (Fig. 3A, B, cf. lane 1' with 2'), suggesting that splicing, enhanced by the strength of the 3'ss, competes with Rev activity.

To address the question of whether a suboptimal 5'ss could compensate for an efficient 3'ss in Rev function we combined a 5'ss of intermediate complementarity to U1 snRNA (-1G3U, Fig. 2A) [28] with the efficient 3'ss A7^+^. In agreement with our previous results, in the presence of A7 this intermediately strong 5'ss led to a 2–3 fold decrease in the amount of RNA (Fig. 3A, cf. lanes 1 and 3, 1' and 3'). However, while the ratio of spliced to unspliced transcripts (Fig. 3A, s/us) was altered only 3-fold, in the presence of A7^+ ^this ratio increased up to 25-fold irrespective of the strength of the 5'ss (Fig. 3). This finding demonstrates that Rev activity is specifically and inversely dependent on the efficiency of the 3'ss A7.

**Figure 4:**
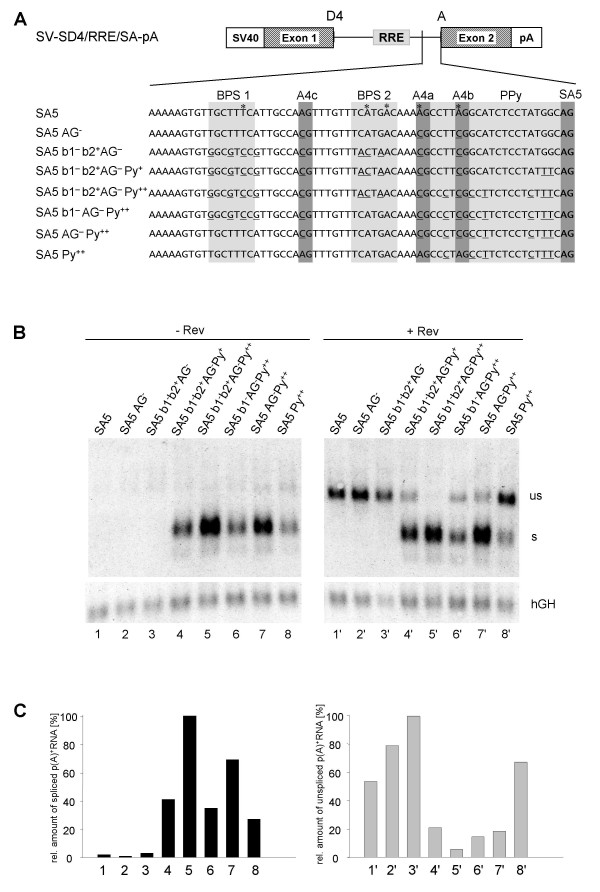
**The strength of the 3'ss competes with Rev responsiveness**. **(A) **Nucleotide sequence of one-intron constructs with mutations in the 3'ss A5. The reported branch point sequences for A5 (grey boxes, asterix indicates the branch point nucleotide), the 3'ss A4c, a, b, A5 (black boxes) and the PPT (hatched) are marked. Mutated nucleotides compared to the wild type are underlined. **(B) **Northern blot analyses of the p(A)^+ ^RNA after transient transfection of HeLa-T4^+ ^cells with one-intron constructs carrying SA5 mutations (cf. Fig.2). **(C) **Diagram of the hGH standardized relative amounts of spliced (left) and unspliced (right) transcripts from (B). The maximal amount was defined as 100%. The numbers below correspond to the lanes of the Northern blot.

**Figure 5:**
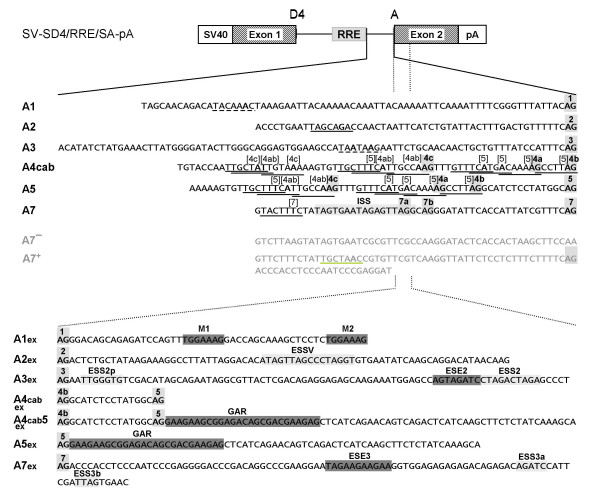
**Schematic drawing of the one-intron splicing reporter**. Diagram of the one-intron construct used for comparison of the HIV-1 3'ss by a splice site swapping strategy. SV40: SV40early promoter, pA: SV40 polyadenylation sequence. RRE: Rev response element. Fragments including the different 3'ss (grey boxes) and branch sites (dashed line: assumed from consensus; underlined: reported BP, numbers are referring to the associated 3'ss, BP A2 [15], BP 4cab and A5 [14,30], BP A7 [14]) were inserted into the cassette. The ISS has been described by [22]. The 3'extended versions of the splice acceptor constructs additionally include the downstream exon sequences with *cis*-acting splicing regulating sequences (M1, M2 [this report]; ESSV [16,64]; ESS2p [18]; ESE2/ESS2 [17,32,43,44,49]; GAR [23,28]; ESE3 [17,21,24,25,33,65]; ESS3a, b [17,21,24,33,66]; splicing silencer (light grey boxes); splicing enhancer (dark grey boxes)).

To determine the sequence requirements of a 3'ss compatible with Rev function in more detail, we constructed a single-intron splice reporter based on a truncated HIV-1 *tat/rev *intron harbouring the RRE (Fig. 4A) and analyzed 3'ss A5 because of its complexity. A5 exhibits a discontinuous pyrimidine stretch and overlaps with the competing alternative 3'ss 4c, 4a and 4b. Moreover, ten BPSs have been experimentally mapped in this region, five of which are associated with splicing at 3'ss A5 [14,30] (see Fig. 5, constructs A4cab and A5). Since the AG-dinucleotides and BPSs can compete for binding of splicing factors we mutated them consecutively (Fig. 4A): First the AG dinucleotides of 3'ss A4c, a and b were changed to CG (AG^-^) to exclude splicing at these positions. Next, the complementarity between the 5' BPS (named BPS1 in Fig. 4A) and U2 snRNA was reduced while the complementarity of the 3' BPS (BPS2) was enhanced (b1^- ^b2^+^). Thirdly, the pyrimidine content was increased from 52% in the wild type 3'ss A5 to 60% (Py^+^) and 72 % (Py^++^), respectively.

Following transient transfection of HeLa-T4^+ ^cells with these constructs the poly(A)^+ ^RNA was analyzed by Northern blot. Neither the mutations of the upstream AGs (Fig. 4B, lane 2) nor of the branch sites (lane 3) led to splicing at the 3'ss A5 but efficiently allowed Rev-dependent detectability of the unspliced transcript (lanes 2' and 3').

Spliced RNA was not detected until the pyrimidine content was further increased (lane 4 and 5). Remarkably, a pyrimidine content of 60% (Py^+^) was still compatible with a low-level of Rev function (lane 4') but in contrast, a highly efficient 3'ss due to a further increase in the pyrimidine content of only 12% (Py^++^) was not (lane 5').

Removing the improvement of BPS2 (SA5 b1^- ^AG^- ^Py^++^) reduced splicing efficiency 3-fold (cf. lane 5 with 6) and concomitantly restored Rev-compatibility (cf. lanes 5' and 6') in spite of the high pyrimidine content. This suggests a comprehensive effect of overall 3'ss strength on Rev activity.

Interestingly, we found no indication that the suboptimal BPS 1 and BPS 2 were competing with each other. Splicing was less efficient than in the construct with an optimal branch site (cf. lane 7 with 5), but enhanced compared to the construct with only one predicted suboptimal branch site (cf. lane 7 with 6). Reconstruction of the AGs of 3'ss A4c, a, b further decreased the level of spliced transcripts (cf. lane 7 with 8) but increased the level of the Rev-dependent unspliced RNA (cf. lane 7' with 8'). In general, the amount of unspliced transcript in the presence of Rev (Fig. 4B and 4C, lanes 1'–8') was inversely proportional to that of the spliced transcript. This confirms our findings shown in Fig. 3, that splicing efficiency driven by the 3'ss competes with Rev function.

### The intrinsic strengths of the HIV-1 splice acceptor sites differ largely

The observation that the efficiency of the 3'ss competes with Rev function implicates that all HIV-1 3'ss should be inefficient to allow the export of unspliced transcripts necessary for virus replication. Indeed, this has been already reported by O'Reilly and coworkers [27] however, at the time of publication the knowledge of HIV-1 splice site regulation by *cis*-acting sequences was rather incomplete.

To differentiate between the contribution to the overall splice site strength of the splice site regulating elements in the 3' exonic sequences and the intrinsic strength of the HIV-1 3'ss we used a splice site swapping strategy and analyzed the HIV-1 3'ss with or without their natural downstream exonic sequences (Aex and A, respectively, Fig. 5). Each 3'ss included the experimentally defined or assumed, by complementarity to U2 snRNA, branch point sequence, the polypyrimidine tract and the AG dinucleotide. Because of their functional and spatial overlap the 3'ss A4c, a and b were experimentally considered as an entity. The 3'ss A6, which is located in the *tat*/*rev *intron, was not included in this analysis because its activity has been described in isolate HIV HXB2 but not in HIV NL4/3 which was used in this study [2,31]. As reference sequences the non functional (A7^-^) and the efficient 3'ss (A7^+^) mutants shown in Fig. 2 and 3 were also included.

**Figure 6:**
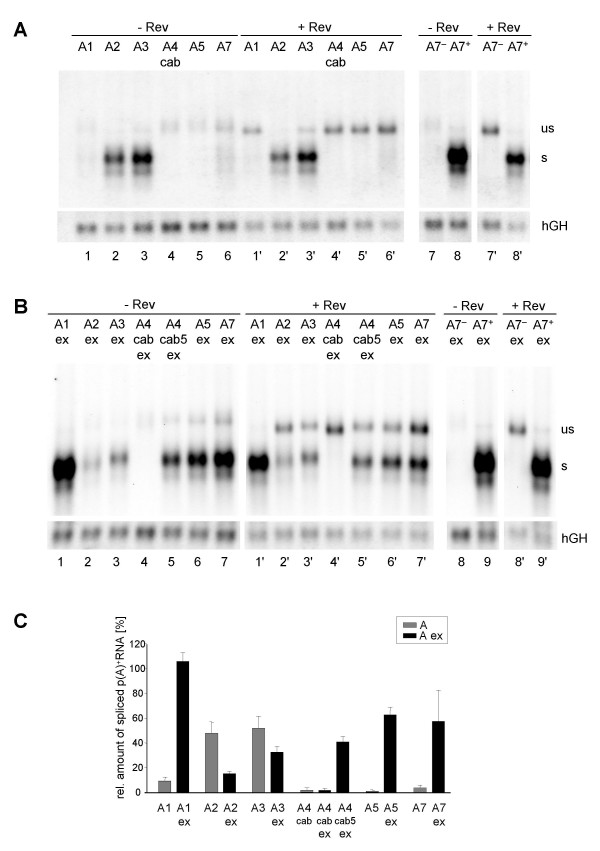
**The strength of the 3'ss competes with Rev responsiveness**. **(A) **Northern blot analysis (cf. Fig. 2) from HeLa-T4^+ ^cells transfected with constructs containing the HIV-1 3'ss in absence of their authentic 3' exon sequences. The particular 3'ss and the co-transfection of a *rev *expressing plasmid (SVcrev) are given above the lanes. The 3'ss A7^- ^and A7^+ ^were used as reference constructs for a nonfunctional and an efficient 3'ss. All lanes were derived from the same Northern blot. **(B) **Northern blot analysis from cells transfected with 3'ss in presence of their authentic 3' exon sequences (ex). All lanes were derived from the same blot. **(C) **Mean values of the relative amounts of spliced transcripts in absence and presence of the 3' exon sequences from three independent experiments, normalized to transcription efficiency (hGH, cf. Fig. 2). The amount of spliced transcripts derived from the construct containing the improved A7^+ ^was defined as 100% (not shown).

Northern blot analyses of these constructs revealed that only 3'ss A2 and A3 led to detection of significant amounts of spliced mRNA in the absence of their natural 3' exonic sequences (Fig. 6A, lanes 2 and 3). Consistent with the results shown in Fig. 3 and 4 these two constructs showed the lowest number of unspliced transcripts in the presence of Rev (Fig. 6A, cf. lanes 2' and 3' with 1', 4'–6'). Thus, these results indicate that 3'ss A2 and A3 are the most efficient core 3'ss, here referred to as the intrinsic efficiency of the 3'ss. For all other 3'ss the intrinsic efficiency was low and significant amounts of unspliced message could be detected in the presence of Rev.

Interestingly, the opposite picture was obtained for the series of constructs where the downstream exonic sequences were included (Fig. 6B and 6C). Compared to their respective intrinsic efficiencies, splicing at A2 and A3 was decreased 3-fold and 1.5-fold in the presence of their downstream exons (Fig. 6C, cf. A2 with A2ex and A3 with A3ex). This is in accordance with the described ESS elements, consisting of three hnRNP A1 binding sites within exon 3 [16], and hnRNP H and hnRNP A/B binding sites within exon 4 [18,32]. Therefore, significant amounts of Rev-dependent, unspliced messages are only detectable if the intrinsic strength of these 3'ss is silenced by their downstream exonic sequence (cf. Fig. 6A, lane 2' and 3' with Fig. 6B, lane 2' and 3').

Since the alternative 3'ss A4c, A4a, A4b and A5 are all in close proximity to each other we tested whether these sites are regulated by the same bidirectional enhancer in exon 5 (A4cab5ex), which also leads to efficient splicing of the flanking 3'ss A5 and 5'ss D4 [23]. Alternatively, additional sequences upstream of this ESE may be sufficient to influence the strength of at least one of the 3'ss A4c, A4a and A4b (A4cabex). The result showed that in the absence of the bidirectional ESE in exon 5 none of these 3'ss could be adequately activated as evident by the absence of any spliced transcript (Fig. 6B, lane 4). Hence, the alternative 3'ss A4c, A4a, A4b and A5 seemed to be moderately activated by the same bidirectional enhancer in exon 5, still allowing Rev-mediated nucleocytoplasmic transport of unspliced transcripts (cf. Fig. 6B, lane 4' with lanes 5' and 6'). Comparison of the amount of spliced transcript from the constructs carrying either the BPS of all 3'ss A4c, A4a, A4b and A5 (Fig. 6C, A4cab5ex) or only the BPS for the 3'ss A4a, A4b and A5 (A5ex) showed a slight increase (30%) in the amount of spliced transcript of the latter. This suggests that competition of the four alternative 3'ss might also contribute to the inefficiency of splicing and that this is also supportive for the Rev-mediated export of the unspliced message.

To date A7 is the only splice site with a known splicing silencer in the intronic region and therefore we cannot distinguish between the impact of the suboptimal PPT and this ISS on the intrinsic inefficiency of this 3'ss (Fig. 6A, lane 6). However, splicing at A7 depends on activation by its flanking downstream sequences carrying the bipartite ESE3/ESS3 regulatory sequence (cf. Fig. 6A lane 6 with Fig. 6B, lane 7) [17,21,25,33]. Thus, in this experimental context, the ESE clearly dominates over the ESS function.

Most strikingly, 3'ss A1 extended by its natural exonic sequence turned out to be the most efficient 3'ss of all (Fig. 6B, cf. lane 1 with 2–7). Even in the presence of Rev, only a very small amount of unspliced message was detected comparable to 3'ss A7^+ ^(Fig. 6B, cf. lane 1' with 9'). Therefore, from these experimental results we conclude that exon 2 contains a strong splicing regulatory element, which has not been identified so far.

These results combined show that, although all HIV-1 3'ss are predicted to be weak on the basis of their intronic sequences, there are distinct differences in their intrinsic splicing efficiency. To co-ordinate both splicing and Rev function the strength of the individual 3'ss is finally regulated by *cis*-regulating ESEs and ESSs in their 3' exons.

### An SF2/ASF-dependent splicing enhancer in exon 2

**Figure 7:**
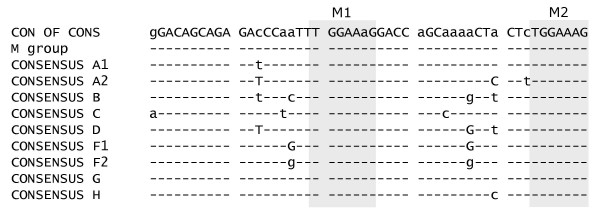
**Alignment of exon 2 of HIV-1 M group consensus sequences**. Exon 2 sequences were obtained from the HIV Sequence Database [67] flanked by A1 and D2. The heptameric sequences (M1 and M2) found to enhance splicing are highlighted by grey boxes.

**Figure 8:**
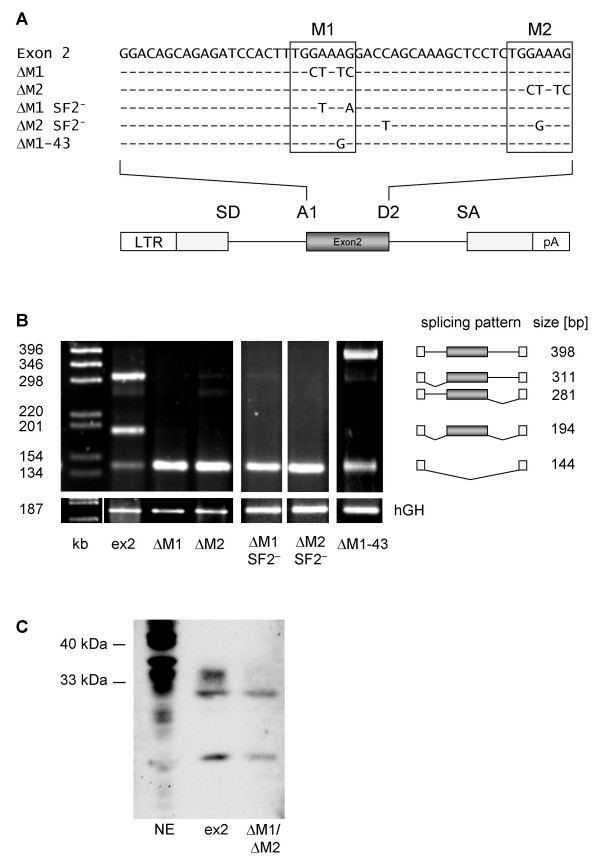
**An ASF/SF2-dependent ESE within exon 2**. **(A) **Sequence of exon 2 (top line) and analyzed mutations. The heptameric sequences (M1 and M2) of the bipartite ESE are boxed and the mutations are given below exon 2 sequence. A schematic drawing of the two-intron minigene with exon 2 as internal exon flanked by A1 and D2 is given below the sequences. **(B) **HeLa-T4^+ ^cells were transfected with LTR ds ex2 (ex2) or its mutations (cf. (A)), cotransfected with SVctat and pXGH5 (hGH) and analyzed by RT-PCR as described in Materials and Methods. **(C) **Pull-down analysis of *in vitro *transcripts of either exon 2 or exon 2 carrying both mutations, M1 and M2 in HeLa cell nuclear extract. The immunoblot was detected with a polyclonal SF2/ASF antibody.

Quantification of the spliced transcripts from three independent Northern blots in the presence and absence of the downstream flanking exonic sequences revealed that the exon 2 sequence improved splicing at the 3'ss A1 about 11-fold (Fig. 6C, cf. A1 and A1ex). A heptameric motif TGGAAAG occurred twice within this relatively short exon of only 50 nucleotides. Moreover, it is conserved in the different strains of the HIV-1 group M (Fig. 7). Consistent with our observation that at least two SR-binding sites are necessary for supporting U1 snRNA binding at 5'ss D4 [34] (Freund and Schaal, unpublished data) we examined whether these heptameric sequences might constitute a bipartite ESE. Therefore, we generated a two-intron minigene construct with exon 2 as the internal exon and mutated either heptamer 1 (M1) or heptamer 2 (M2) (Fig. 8A). RT-PCR analysis of the transcripts following transient transfection of HeLa-T4^+ ^cells revealed that mutating either of one of these heptamers totally abolished exon 2 inclusion (Fig. 8B, cf. lane ex2 with Δ M1 and Δ M2). Thus, this heptameric motif most likely constitutes a key element of an ESE in exon 2. Furthermore, it confirms our hypothesis that at least two putative binding sites are necessary to define a functional enhancer. Since GAAAGGA was predicted to bind SF2/ASF by ESEfinder [35] we analyzed SF2/ASF-binding by pull-down and subsequent Western blot analysis using a polyclonal antibody against SF2/ASF. As shown in Fig. 8C immunoblot analysis of proteins from HeLa nuclear extracts pre-incubated with either RNA of *in vitro *transcribed exon 2 or exon 2 carrying mutations Δ M1 and Δ M2 revealed that the introduced mutations led to reduced reactivity of a polyclonal SF2/ASF antibody with a band of the corresponding molecular mass of SF2/ASF-binding sites. To confirm the SF2/ASF-dependent exon 2 recognition two additional mutations were analyzed which were predicted by ESEfinder to specifically abolish SF2/ASF-binding. As expected these two constructs (Δ M1 SF2^-^, Δ M2 SF2^-^) led to a complete lack of exon 2 recognition (Fig. 8B) supporting the observation that M1 and M2 represent the SF2/ASF-dependent exon 2 splicing enhancer. Since we were interested in analyzing this newly identified ESE within an infectious molecular clone we set out to test for a silent point mutation predicted to specifically inactivate this enhancer. Based on computer analysis only one mutation was found that fulfilled the desired criterion (Δ M1 – 43). The mutation Δ M1 – 43 resulted in a slightly reduced loss of exon 2 recognition compared to the other mutations (Fig. 8B). The most obvious difference was the appearance of a comparable amount of unspliced transcript. Nevertheless, this result confirmed that the Δ M1 – 43 mutation affected exon 2 recognition.

Therefore, we inserted the Δ M1 – 43 mutation into the molecular clone NL4/3 and investigated its effect on viral replication in PM1 cells. Unexpectedly, no difference in the replication kinetic was observed throughout the infection period of up to 10 days (data not shown) suggesting that Δ M1 – 43 could not dramatically impair viral gene expression. However, analyzing the viral mRNA of the infected cells at day 5 post-infection by RT-PCR using primers specific for the upstream region of the HIV-1 genome revealed a different splicing pattern for the non-coding leading exons (Fig. 8A). As observed within the splicing reporter, the presence of the Δ M1 – 43 mutation led to a significant loss of exon 2 recognition (cf. band 1.2.4 in lanes NL4/3 and Δ M1-43) confirming that the newly identified ESE within exon 2 supports exon definition within the context of the viral genome. Furthermore, the lack of exon 2 recognition was accompanied by an increase in exon 3 recognition (cf. band 1.3.4 in lanes NL4/3 and Δ M1-43). These results support recent findings that the optional leader exons might not play a direct role in viral gene expression [36]. The most abundant detectable spliced isoform corresponded to a 1.4 transcript which was not expected to be affected by the mutation. However, most surprisingly, the amount of *vif *mRNA was also not altered by the Δ M1 – 43 mutation (cf. band 1.2l in lanes NL4/3 and Δ M1-43), demonstrating that this mutation exclusively impaired exon 2 definition but not intron (D1 – A2) definition. To confirm these results functionally we compared the replication kinetics of NL4/3 and NL4/3 Δ M1-43 in the *vif *non-permissive and permissive cell lines CEM and CEM-SS respectively. Consistent with the PM1 infection experiments no difference in the viral replication kinetics could be observed between CEM and CEM-SS (data not shown) indicating that the level of *vif *expression is not impaired by the Δ M1 – 43 mutation during the infectious experiment up to 16 days.

**Figure 9:**
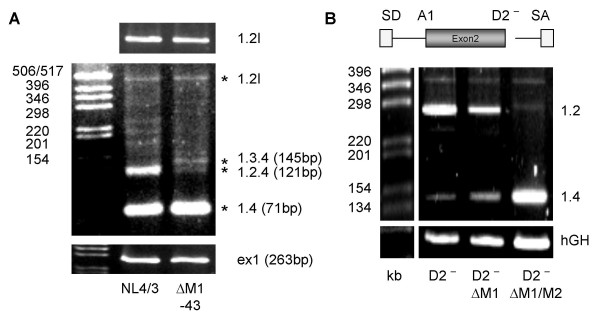
**Mutation of M1 by a single, silent point mutation does not alter *vif *mRNA expression within an infectious molecular clone**. HIV-1 splicing pattern of PM1 cells infected with NL4/3 or NL4/3 carrying a silent point mutation within M1 (ΔM1-43, cf. Fig. 8A). The upper part shows an RT-PCR amplification for the *vif *1.2I mRNA (the letter I denotes incompletely spliced or intron-containing mRNA) using primer pair #1544/#2183. The middle part was obtained using primer pair #1544/#1542. The stars indicate that these bands were isolated from the gel and confirmed by sequencing. To compare the total amount of HIV-1 transcripts within both mRNA preparations a separate RT-PCR was performed using primer pair #2335/#480 amplifying exon 1 sequence. **(B) **RT-PCR analysis of RNA from HeLa-T4^+ ^cells transfected with the minigene lacking D2 (cf. figure legend 8B).

To further investigate the apparent discrepancy between the transient transfection experiment using the splicing reporter (cf. Fig. 8B, lane Δ M1-43) and the infection experiments we specifically analyzed the effect of the Δ M1 mutation on splice site usage of A1 in the splicing reporter by excluding exon definition through deletion of 5'ss D2. Similar to the infection experiments 3'ss A1 usage involving in intron definition still occurs in the presence of Δ M1 mutation although to a somewhat lesser extent (Fig. 9B, D2^- ^Δ M1). In contrast, mutating both heptameric sequences (Fig. 9B, cf. D2^- ^Δ M1 with D2^- ^Δ M1/2) resulted in a total failure of 3'ss A1 recognition. Thus, the intron-containing Rev-dependent *vif *mRNA is less dependent on the strength of the bipartite ESE (if defined through the number of SR binding sites).

## Discussion

Previous mutational analyses of HIV 5'ss D4 revealed that its splice function was inessential for Rev function but that increased binding of U1 snRNA to this region correlates with increased steady state levels of spliced as well as intron-containing subgenomic HIV-1 transcripts [28,37,38]. Therefore, we investigated the specific influence of the HIV-1 3'ss on RNA steady state levels and Rev responsiveness. In a subgenomic *env *construct we found that inactivation of 3'ss A7 and the upstream cryptic sites A7a and A7b totally abolished splicing of the *tat/rev *intron, without affecting the number of unspliced Rev-dependent transcripts. Thus, Rev function requires neither a splicing-competent 5' nor 3'ss. This is in accordance with earlier findings that Rev can act independently from splicing provided that U1 snRNA can bind the *env *RNA to protect it from degradation [39,40]. It also confirms that stabilization of the HIV-1 RNA upon U1 snRNA binding to the 5'ss is independent of late spliceosome formation. On the other hand an increase in 3'ss A7 splicing efficiency (inferred from a greater amount of detectable spliced mRNA in the absence of Rev), corresponded with decreased amounts of unspliced Rev-dependent mRNAs. Consistent with this, the rate limiting step determining the kinetic of the splicing reaction seems predominantly determined by the strength of 3'ss. This determines the use of the pre-mRNAs as a splicing substrate or as a target for Rev-dependent nuclear export. This model is supported by the finding that slightly reducing the strength of the 5'ss only led to moderate differences in the ratio of spliced versus unspliced subgenomic transcripts while mutating the 3'ss altered the ratio at least 10-fold (Fig. 3A).

Efficient 3'ss compete with Rev function but retroviral replication requires an equilibrium between splicing and nuclear export of intron-containing transcripts and therefore the low efficiency of the 3'ss is a key factor for viral replication. Under such suboptimal conditions a slow down of the first transesterification reaction is likely [14,41,42]. Nevertheless, a prediction of 3'ss efficiency based only on the evaluation of the sequence by available algorithms is still not reliable in all cases due to the complex interplay of the U snRNAs and proteins involved in 3'ss recognition. An experimental assessment of the splicing efficiency of the HIV-1 3'ss was performed by O'Reilly and coworkers [27]. In a heterologous β-globin/HIV-1 construct they evaluated the relative efficiencies of the HIV-1 3'ss compared to the β-globin 3'ss A1 which was used as a reference for an efficient 3'ss. The outcome of this study was a relative homologous clustering of the HIV-1 3'ss between 40% and 60% splicing efficiency with exception of 3'ss A1 (in the original publication referred to as 3'ss A2, see also Fig. 1) which showed a significantly lower efficiency (26%). Unfortunately, at that time the relevance and multitude of *cis*-acting sequences regulating alternative splicing in the HIV-1 transcripts are just starting to be unfolded. Therefore, only the downstream exonic *cis*-acting sequence involved in regulation of 3'ss A7 [21,33] was completely included in the investigated fragment (101 nt 3' of the intron/exon border). However, in the past, it had become evident that *trans*-acting factors are involved in constitutive as well as alternative splicing and that almost all HIV-1 exons and also some intron sequences include splicing enhancers and/or silencers [16-18,21-23,28,33,43,44] (see Fig. 1, for recent reviews see [5,45]. Hence, from this apparent ubiquitous presence of *cis*-acting sequences the question arose as to how the classical elements defining a canonical splice site (BPS, PPT, AG-dinucleotide) and the *cis*-acting elements contribute to the overall strength of the different HIV-1 3'ss and their response to Rev. To evaluate the impact on the intrinsic strength of the 3'ss, i.e., the intronic sequence versus the *cis*-acting, exon-located enhancer and silencer elements, we compared the efficiency of the HIV-1 3'ss A1, 2, 3, 4cab and 5 in the presence and absence of their natural downstream exonic sequences in a splice site swapping strategy.

Our comparison of the HIV-1 3'ss with an optimized 3'ss as an internal reference with almost no response to Rev, revealed significant variation in the strength of the viral 3'ss with relative splicing efficiencies from 1% to 52% in the absence and 15% to 106% in the presence of their natural 3' sequences. Based on these data we grouped the HIV-1 3'ss into two different categories. The first category includes 3'ss A1, A4cab, A5 and A7 which were all but inactive in the absence of their 3' exonic sequences. Their 3' sequences had an overall stimulatory effect on splicing efficiency at these sites. This is especially interesting for the increase in splicing efficiency at A7 (from 4 to 57%) since this 3' exon includes both a splicing enhancer and a silencer [21,25,43] that has been shown to compete with ASF/SF2-binding at the ESE and hnRNPA1 binding at the ESS [24,26,46]. Thus, different ratios of ASF/SF2 and hnRNP A1 in different cells may lead to differences in the activation of A7. Under our experimental conditions, however, a dramatic influence of the ESS on the strength of A7 was not detectable (data not shown). Hence, the ESE was clearly dominant over the ESS function. Additionally, we identified a new *cis*-acting sequence within exon 2, which profoundly increased splicing efficiency at 3'ss A1. This regulatory element includes two TGGAAAG heptameric sequences which constitute key elements of a bipartite ESE and support our observation that a functional enhancer seems to be defined by at least two individual binding sites [34]. The identification of ASF/SF2 as the respective splicing regulatory protein is in agreement with the finding, that overexpression of ASF/SF2 stimulated splicing at site A1 in HIV-1 pNL4/3 transfected 293T cells [47].

The second group of 3'ss, A2 and A3, showed approximately 50% splicing efficiency in the absence of exon sequences and a 2–3 fold decrease in efficiency in the presence of exon sequences, consistent with published ESS sequences in exon 3 (ESSV) and 4 (ESS2p, ESS2) (see Fig. 1) [16-18,43,48]. Despite the presence of these ESSs usage of both 3'ss could also be stimulated *in vivo *following overexpression of ASF/SF2 [47] and *in vitro *upon addition of recombinant SC35 [49] disclosing the ambivalence of HIV-1 3'ss regulation. A reason for the restrictive control of 3'ss A2 and A3 might be the cytotoxicity of Tat [50] and Vpr [51] which are translated as first reading frames of the appropriate mRNAs.

Comparing the two splice site groups we noticed that the pyrimidine content of the PPT was highest in 3'ss A2 and A3 (65 and 69%) and lower in the other 3'ss (40% up to max. 62%). This encouraged us to analyze the contribution of the intronic sequence elements, i.e., BPS, PPT and the AG-dinucleotide, to the efficiency of the HIV-1 3'ss. To this end, taking the A5 sequence as an example, we increased the pyrimidine content stepwise from 52/55% in the wild-type sequence to 60% (Py^+^) and 72% (Py^++^) and combined it with an improved complementarity of the branch site to U2 snRNA and removal of competing AG dinucleotides A4c, a, b (Fig. 4). Simultaneous improvement of these elements led to enhanced splicing at A5 with no response to Rev. Moreover, if only one of the elements was altered exclusively the increase of the pyrimidine content to 72% but not to 60% led to enhanced splicing at A5 (data not shown). The altered branch point and removed AGs were only able to increase splicing in the presence of 60% or more pyrimidines. These findings show that the differences in the pyrimidine content of the HIV-1 3'ss only as a first approximation could explain their different splicing efficiencies.

**Figure 10:**
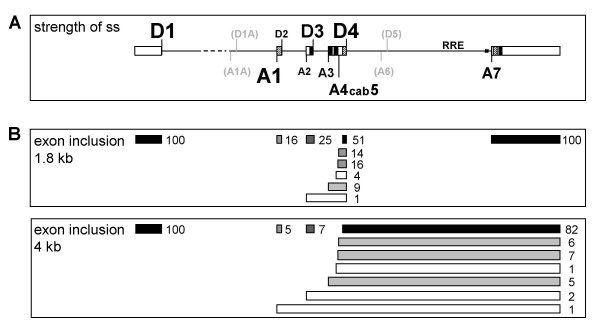
**Schematic overview of HIV-1 splice sites and their strength**. **(A) **Schematic drawing of the HIV-1 pre-mRNA and the distribution of the splice sites. The relative strength of the splice sites, based on splice site swapping strategies in this and previous publications [27,29], is represented by the size of the letters. D5 and A6 (grey) are marked for better orientation but they are not used in HIV-1 NL4/3 and therefore their relative strength was not tested. D1A and A1A are recently published splice sites preferentially involved in RNA stabilization [6]. ESEs (hatched) and ESS/ISS (black) are marked. **(B) **Schematic drawing of the exon structure in the 1.8 kb and 4 kb HIV-1 RNA classes. The numbers represent the relative incidence of exon inclusion [%] calculated from previously published RT-PCR analyses [2]. Differences to 100% in the total amount of the possibilities of overlapping exon recognition are due to rounding errors. The darker the colour of the exons the more efficiently they are included in the alternatively spliced HIV-1 transcripts.

As previously determined experimentally for all HIV-1 5'ss [28,29] and here for all HIV-1 3'ss, there is no strict alternation between strong 5'ss and weak 3'ss as discussed recently [52]. On the contrary, D1 and A1 are the most efficient splice sites defining the first intron. Nevertheless, this splice site pair is not the most frequently recognized pair (based on the frequencies of only 16% exon 2 inclusion and 1% *vif *mRNA, Fig. 10B) most likely due to the intrinsically weakest 5'ss D2 which opposes cross-talk of the splice site pair across exon 2. This suggests that, although harboring a bipartite ESE HIV-1 exon 2 will be efficiently included only if the intrinsic strength of D2 is increased. Indeed, this has been shown most recently resulting in decreased virus production [36]. However, our finding that a switch in splice site pair recognition can crucially depend on exonic *cis*-acting regulatory sequences supports the possibility that Rev by interacting with *trans*-acting splicing regulatory proteins could switch cross-talk of splices site pairs. Alternatively, by interacting with *trans*-acting splicing regulatory proteins, Rev could functionally substitute for an ESE. Consistent with this, Rev has been found to bridge p32 which co-purifies with ASF/SF2 to the RRE thereby possibly stabilizing the interaction of U1 snRNP with the 5'ss and arresting further spliceosome formation [53]. This model provides an explanation for our finding that partially inactivating the ESE (Δ M1 – 43 mutation) did not affect processing of the Rev-dependent 1.2I *vif *mRNA but specifically leads to loss of exon 2 recognition within the Rev-independent class of mRNAs.

Although it seems that HIV-1 splice site regulation is highly complex as outlined here (without even addressing the possible impact of secondary structures or superordinated hairpin structures on splice sites usage [54,55]) it is this complexity that provides a possibility to selectively inhibit HIV-1 splice site usage as a potential treatment strategy without cellular side effects [56,57].

## Methods

### Oligonucleotides

Oligonucleotides were synthesized and purified as previously described [58]. Sequences are given in Tab. 1.

**Table 1:** Oligonucleotides used throughout these experiments

**Oligo no.**	**sequence**
#139	5'-CCCAAGCTTTCTAGACTCGAGCTACAAAATCCTTTC
#377	5'-CTGAAGCGCGCACGGCAAGAGGCGAGGGGAGGCGACTG
#480	5'-GCGCGCTTCAGCAAGC
#481	5'-GCGCGCACGGCAAGA
#559	5'-CTTTACGATGCCATTGGGA
#626	5'-TGTCTTAAGTATAGTGAATCGCGTTCGCCAAGGATACTCACCA
#628	5'-CCCCTCGGGATTGGGAGGTGGGTTTGGAAGCTTAGTGGTGAGTATCC
#796	5'-TGAGGTTACCGTTCTTTCTATT
#797	5'-GATCCCGGGATCCTCGGGATTGGGAGGTGGGTCTGAAAAGAAAGAGGAGAATAACCTTGACGAACACGGTTAGCAATAGAAAGAAC
#798	5'-TGAACTAGTAGGTTTAAGAATAGTTTTTGCTGTTCTTTCTATT
#800	5'-TGAGGTTACCGTACTTTCTATAGTGAAT
#918	5'-GCTATGTCGACAAGGAGCTGCAGATCGATGAATTCGATACTTACCAGTCGCCTCCCCTC
#931	5'-GCTATGTCGACACCCAATTCTGAAACGATAATGGTGAATATCCCTGCCTAACTCTATTCACTATA
#932	5'-ATCCTGCAGAATAGTTTTTGCTGTACTTTCTATAGTGA
#936	5'-ACTGGTTACCACATATCTATGAAACT
#939	5'-ACAGGATCCATCCCCGGGCTGAAATGGATAAACA
#940	5'-ACTGGTTACCTGTACCAATTGCTATT
#941	5'-ACAGGATCCATCCCCGGGCTAAGGCTTTTGTCAT
#942	5'-ACTGGTTACCAAAAAGTGTTGCTTTC
#943	5'-ACAGGATCCATCCCCGGGCTGCCATAGGAGATGC
#945	5'-ACAGGATCCATCCCCGGGCTGAAAAACAGTCAAA
#946	5'-ACTGGTTACCACCCTGAATTAGCAGA
#947	5'-ACAGGATCCATCCCCGGGCTGTAATAAACCCGAA
#948	5'-ACTGGTTACCTAGCAACAGACATACA
#985	5'-GATGGATCCCGGGCTCGGGATTGGGAG
#986	5'-TGAGGTTACCGTCTTAAGTATA
#1065	5'-ATCCCCGGGCTGAAACGATAATGGTGA
#1088	5'-ACAGGATCCATCCCCGGGTGCTTTGATAGAGAAG
#1089	5'-ACAGGATCCATCCCCGGGCTTTCCAGAGGAGCTT
#1091	5'-ACAGGATCCATCCCCGGGCTTGTTATGTCCTGCT
#1183	5'-CTAGAATTCAGCAACAGACATACA
#1224	5'-TCTTCCAGCCTCCCATCAGCGTTTGG
#1225	5'-CAACAGAAATCCAACCTAGAGCTGCT
#1388	5'-TGAGGTTACCAAAAAGTGTTGCTTTCATTGCCACGTTTGTTTCATGAC
#1389	5'-ATCCCCGGGCTGAAAGAGGAGAAGGCGAGGGCGTTTGTCATGAAAC
#1481	5'-ATCCCCGGGCTGAAAGAGGAGAAGGCTAGGGCTTTTGTCATGAAAC
#1482	5'-TGAGGTTACCAAAAAGTGTGGCGTCCGTTGCCACGTTTGTTTCATGAC
#1483	5'-ATCCCCGGGCTGCCATAGGAGATGCCGAAGGCGTTTGTCATGAAAC
#1484	5'-ATCCCCGGGCTGCCATAGGAGATGCCGAAGGCGTTTGTTAGTAAAC
#1486	5'-ATCCCCGGGCTGAAATAGGAGATGCCTAAGGCTTTTGTCATGAAAC
#1492	5'-ACAGGATCCATCCCCGGGAGGGCTCTAGTCTAGG
#1542	5'-CACCTTCTTCTTCTATTCCTT
#1544	5'-CTTGAAAGCGAAAGTAAAGC
#1586	5'-TGAGGTTACCAAAAAGTGTGGCGTCCGTTGCCACGTTTGTTTACTAAC
#1587	5'-ATCCCCGGGCTGAAAGAGGAGAAGGCGAGGGCGTTTGTTAGTAAAC
#1590	5'-ATCCCCGGGCTGAAAGAGGAGAAGGCGAGGGCGTTTGTCATGAAAC
#1591	5'-TGAGGTTACCAAAAAGTGTTGCTTTCATTGCCACGTTTGTTTCATGAC
#1592	5'-ATCCCCGGGCTGAAATAGGAGATGCCGAAGGCGTTTGTTAGTAAAC
#1913	5'-TACTGCAGTACTTTTATGTCACTATTATCTTGTATTACTACTGCCCCTTCACCTTTCCAGAGGAGCTTTGCTG
#1913a	5'-CTACTGCAGTACTTTTATGTCACTATTATCTTGTATTACTACTGCCCCTTCACCTTTCCAGAGGAGCTTTGCTGGTCGATAGCAAACTGGATCTCTG
#1913b	5'-CTACTGCAGTACTTTTATGTCACTATTATCTTGTATTACTACTGCCCCTTCACGATAGCAGAGGAGCTTTGCTG
#2183	5'-GGTCAGGGTCTACTTGTGTGC
#2335	5'-GGGTCTCTCTGGTTAGACCAG

### Recombinant plasmids

SVcrev was constructed by cloning the *Eco*RI-*Xho*I fragment from pUHcrev [59] into pSVT7. The SV40 early *env *expression vector SV E/X tat^- ^rev^- ^contains the *Eco*RI-*Xho*I fragment of pNLA1 [60], a cDNA derivative of pNL4/3. The 5' ss mutations were constructed as previously described [28]. To introduce the mutations A7^- ^and A7^+ ^in 3'ss A7, overlapping oligos were PCR amplified (A7^-^: #626, #628; A7^+^: #797, #798), the PCR products were restricted with *Ava*I/*Afl*II (A7^-^) and *Spe*I/*Xma*I (A7^+^), respectively, and cloned into the appropriate vector backbones.

The one-intron constructs (SV-SD/RRE/SA-pA) and mutations in the 5'ss were constructed as previously described [28]. Mutations in the 3'ss were introduced by swapping the *Bst*EII-*Xma*I fragment except for A7ex which was cloned as a *Bst*EII-*Bam*HI fragment. For A7^+ ^overlapping oligos (#796/#797) were amplified. For A7 ^- ^a fragment was amplified using primer pair #985/#986 and plasmid SV E/X tat ^-^rev ^- ^SA7 ^- ^as template. HIV 1 3'ss A1 sequence was amplified using pNL-gpt (kindly provided by Valerie Bosch, ATV-DKFZ Heidelberg) as template both with 5' primer #948 and 3' primer either #947 (A1) or #1089 (A1ex). All other HIV 3' ss were amplified using pNLA1 as template and primers #946 and either #945 (A2) or #1091 (A2ex), #936 and #939 (A3) or #1492 (A3ex), #940 and #941 (A4cab) or #943 (A4cab5) or #1088 (A4cab5ex), #942 and #943 (A5) or #1088 (A5ex), #800 and #1065 (A7) or #139 (A7ex). The A5 mutations were constructed by introducing PCR products from overlapping oligos (A5 AG ^-^: #1591, #1483; A5 b1^-^b2^+^AG ^-^: #1586, #1484; A5 b1^-^b2^+^AG ^-^Py^+^: #1586, #1592; A5 b1^-^b2^+^AG ^-^Py^++^: #1586, #1587; A5 b1^-^AG ^-^Py^++^: #1482, #1389; A5 AG ^-^Py^++^: #1388, #1590). For A5 Py^++ ^(#942, #1481) and A5 Py^+ ^(#942, #1486) pNLA1 was used as a PCR template.

To clone the three-exon-two-intron minigene reporter construct LTR ds ex2 two PCR fragments containing a 5'ss (#377, #918; *Bss*HII-*Sal*I) and a 3'ss (#931, #932; *Pst*I-*Sal*I) were inserted into LTR 1.4tatCAT, a vector coding for transcripts with the native HIV-1 tat 1.4 mRNA leader sequence [61], to generate LTR SD SA tatCAT.

Exon 2 including flanking intronic sequences of pNL-gpt was PCR-amplified with primers #1183 and #1913 and cloned into LTR SD SA tatCAT via *Eco*RI and *Pst*I. Similarly, to insert the mutations of heptamer 1 and 2 the 3' PCR primer was substituted for #1913a (LTR ds ex2 hept.1) and #1913b (LTR ds ex2 hept.2) respectively.

All plasmid sequences can be obtained on request.

### Cell culture, transfection and Northern blot analysis

HeLa-T4^+ ^cells [62] were transfected with FuGENE™ 6 (Roche Molecular Biochemicals) and total RNA was prepared 30 h after transfection by a modified guanidinium isothiocyanate protocol [63] using RNA-clean (Hybaid-AGS, Heidelberg). The poly(A)^+ ^RNA from 80–100 μg total RNA was isolated with Dynabeads^® ^oligo(dT)_25 _(Dynal, Oslo), subjected to electrophoresis on a 1.2% agarose-1% formaldehyde-gel, and blotted onto a positively charged nylon membrane (Roche Molecular Biochemicals). After UV crosslinking (0.5 J/cm^2^), the membrane was hybridized with digoxigenin-(DIG) labeled antisense RNA probes in a buffer containing 50% (v/v) formamide, 5 × SSC, 50 mM sodium phosphate (pH 7), 0.1% (w/v) N-lauroylsarcosine, 7% (w/v) SDS, 2% (w/v) blocking reagent (Roche Molecular Biochemicals), 50 mg yeast RNA/L at 68°C. To monitor transfection efficiency and RNA loading, the membrane was hybridized with a DIG-labeled antisense RNA probe specific for exon 5 of human growth hormone (hGH, expressed from cotransfected plasmid pXGH5 as a transfection control). HIV-specific RNA was detected by a DIG-labeled antisense RNA probe specific for the 3' end of *env *(LTRcenvpA^-^, nt 8648–8887) and detection with anti-Digoxigenin-AP-Fab fragments (50 mU/ml; Roche Molecular Biochemicals) and chemiluminescence substrate (250 μM CDP-Star™; Roche Molecular Biochemicals) as previously described [28]. Quantification was done with the Lumi-Imager F1 (Roche Molecular Biochemicals) and the LumiAnalyst™ 3.1 software.

### RT-PCR assay

Isolation of total RNA was performed using a modified guanidinium isothiocyanate protocol [63]. Cells were washed twice with 2 ml of PBS and cell lysis was performed with 500 μl of buffer D [4 M guanidinium-isothiocyanat, 25 mM Na-Citrat pH 7, 0,5% N-Laurylsarkosin]; 7.6 μl of 2-mercaptoethanol, 50 μl of 3 M sodium acetate (pH 4), 500 μl of phenol and 100 μl of a chloroform-isoamyl alcohol mixture (24:1) were added and mixed for 15 s. After incubation on ice for 15 min, phases were seperated by centrifugation (10,600 × g, 4°C, 20 min). RNA was precipitated in 1 volume of isopropanol overnight. After centrifugation (10,600 × g, 4°C, 20 min) the RNA pellet was washed twice with 70% ethanol and dissolved in 10 μl of DMDC-ddH2O. Prior to reverse transcription, 4 μl of RNA samples were subjected to DNase I digestion using 10U DNase I (Roche Molecular Biochemicals) with 50 mM Tris (pH 7.5) and 10 mM MgCl_2 _in a total volume of 10 μl at room temperature for 1 h. After DNase I inactivation at 80°C for 10 min, 4.5 μl of the DNase digested RNA samples were reversed transcribed with 200U SuperScript III RNase H-Reverse Transcriptase (Invitrogen) according to the manufacturer's protocol using 0.375 mM oligo(dT)_15 _(Roche Molecular Biocemicals) or 0.02 μM sequence-specific oligo #1542 as primer. As a negative control for the remaining plasmid DNA contamination of each sample, a second assay was performed as described above but replacing reverse transcriptase with ddH_2_O.

PCR was carried out with 1.25U AmpliTaq (Applied Biosystems) in a total volume of 50 μl according to the manufacturer' protocol in a Robocycler Gradient 96 Temperature Cycler (Stratagene). All primers were used at a final concentration of 0.2 μM. Spliced and skipped RNA was detected with the primer pair #1544/#1542 and hGH mRNA with primer pair #1225/#1224. Prior to PCR the cDNA reaction mixture was denatured at 94°C for 3 min. To determine the linear PCR-amplification range allowing a semi-quantitative estimation of the relative abundance of pSV-1-env and hGH mRNA, a preliminary PCR test series was carried out using the same cDNA sample but varying the PCR cycle numbers between 15 and 30 [94°C, 0.5 min; 52°C (pSV-1-env) and 56°C (hGH), respectively, 1 min; 72°C, 1 min]. The reactions were completed with a final elongation step of premature amplified products at 72°C for 10 min. Accordingly to the obtained results, PCR analysis was performed with 26 cycles for pSV-1-env as well as hGH PCR amplification.

PCR products were separated on 6% non-denaturating polyacrylamide gels, stained with ethidium bromide (10 min) and visualized with the Lumi-Imager F1 (Roche Molecular Biochemicals).

### Pull-down Assay of ASF/SF2

Periodat-labeled *in vitro *transcripts (1 nmol) of *Eco*47lII-linearized T7 Ex2 and T7 Ex2 Δ M1/Δ M2 plasmids carrying mutations of the heptameric motive were prepared using T7-MEGAshortscript™ (Ambion) and 0.1 M sodium m-periodat. The binding reaction to the adipic acid dihydrazide agarose (Sigma) was performed over night in 0.1 M NaOAc pH 5.0. Complex formation was performed in a 650 μl reaction volume containing 500 μl of HeLa nuclear extract (Cell Culture Center, Belgium) and 150 μl buffer D (20 mM HEPES-KOH pH 7.9, 100 mM KCl, 20% glycerol, 0.2 mM EDTA, 0.5 mM DTT) by incubation at 30°C for 30 min. After washing five times in 1 ml of buffer D the complexed RNAs were eluted by incubating the beads in 60 μl of 2 × sample buffer, heating at 90°C for 10 min, followed by fractionation on an 7% denaturing polyacrylamide gel. The proteins were blotted onto a PVDF membrane (Immobilon™ P, pore size 0.45 μm; Millipore) by electroblotting with 70V in transfer buffer (200 mM glycine, 25 mM Tris, 20% methanol) for 1 h. Blots were blocked o/n in PBS with 10% bovine serum albumin (BSA), 10% Tween^®^-20. Protein detection was performed in PBS, 1% BSA, 1% Tween^®^-20, with a goat polyclonal antibody raised against a peptide mapping near the C-terminus of SF2/ASF of human origin (Santa Cruz Biotechnology, Inc. C-19:sc-10255) for 1 h, washed three times, incubated with a horseradish peroxidase conjugated AffiniPure donkey anti-goat IgG (Jackson ImmunoResearch Laboratories, Inc. #705-035-147), washed four times, rinsed with water and visualized by a chemiluminescence detection system (ECL™-system and ECL™ hyperfilm, Amersham; Super Signal^® ^ultra, Pierce).

### HIV-1 infection experiments

Cell cultures were maintained in RPMI 1640 medium containing 10% fetal calf serum (Pansystems GmbH) and antibiotics (penicillin and streptomycin). Viral stocks were prepared by transfecting 293T cells with provirus expression vectors, followed by ELISA (Innotest HIV p24 Antigen mAb; Innogenetics N. V.) of culture supernatants for p24 content. For HIV-1 infection, 5 × 10^6 ^PM1, CEM or CEM-SS cells were resuspended in 500 μl culture medium and incubated at 37°C for 3 hours with 100 ng of each HIV-1 viral stock (HIV-1 NL4/3 wild type and ΔM1-43). After infection, cells were washed twice with PBS without Ca^2+ ^and Mg^2+ ^and further cultured in 5 ml medium for another 5 days. Subsequently, total RNA was isolated using Trizol reagent according to the protocol of the manufacturer (Invitrogen).

## Competing interests

The author(s) declare that they have no competing interests.

## Authors' contributions

SK and MO performed the cloning work. Northern blot analyses were carried out by SK, RT-PCR and pull-down by MO. All infectious experiment were designed and conducted in a P3 facility by IH and JH. JK conceived optimization of A7. HS devised and coordinated the study. SK and HS drafted the manuscript. All authors read and approved the final manuscript.
